# Limitations of predicting microvascular invasion in patients with hepatocellular cancer prior to liver transplantation

**DOI:** 10.1038/srep39881

**Published:** 2017-01-06

**Authors:** Michał Grąt, Jan Stypułkowski, Waldemar Patkowski, Emil Bik, Maciej Krasnodębski, Karolina M. Wronka, Zbigniew Lewandowski, Michał Wasilewicz, Karolina Grąt, Łukasz Masior, Joanna Ligocka, Marek Krawczyk

**Affiliations:** 1 Department of General, Transplant and Liver Surgery, Medical University of Warsaw, Poland; 2 Department of Epidemiology, Medical University of Warsaw, Poland; 3 Hepatology and Internal Medicine Unit, Department of General, Transplant and Liver Surgery, Medical University of Warsaw, Poland; 4 Second Department of Clinical Radiology, Medical University of Warsaw, Poland

## Abstract

Microvascular invasion (MVI) is well known to negatively influence outcomes following surgical treatment of hepatocellular cancer (HCC) patients. The aim of this study was to evaluate the rationale for prediction of MVI before liver transplantation (LT). Data of 200 HCC patients after LT were subject to retrospective analysis. MVI was present in 57 patients (28.5%). Tumor number (p = 0.001) and size (p = 0.009), and alpha-fetoprotein (p = 0.049) were independent predictors of MVI used to create a prediction model, defined as: 0.293x(tumor number) + 0.283x(tumor size in cm) + 0.164xlog_e_(alpha-fetoprotein in ng/ml) (c statistic  =  0.743). The established cut-off (≥2.24) was associated with sensitivity and specificity of 72%. MVI was not an independent risk factor for recurrence (p = 0.307), in contrast to tumor number (p = 0.047) and size (p < 0.001), alpha-fetoprotein (p < 0.001) and poor differentiation (p = 0.039). Recurrence-free survival at 5 years for patients without MVI was 85.9% as compared to 83.3% (p = 0.546) and 55.3% (p = 0.001) for patients with false negative and true positive prediction of MVI, respectively. The use of both morphological and biological tumor features enables effective pre-transplant prediction of high-risk MVI. Provided that these parameters are combined in selection of HCC patients for LT, pre-transplant identification of all patients with MVI does not appear necessary.

Liver transplantation provides superior results in well-selected patients with liver cirrhosis and hepatocellular cancer (HCC)^1^. Despite the advantages, its wide use remains limited by the relative shortage of organs procured from deceased donors^2^. Selection of patients based on the Milan criteria is associated with 5-year survival and recurrence rates of approximately 60–80% and 10–20%, respectively^3^,^4^,^5^. Although they remain the benchmark for patient selection, the long-term post-transplant outcomes of HCC patients are generally lower than that observed for patients with benign indications^6^. Nevertheless, authors from numerous transplant centers across the globe advocate expansion of the Milan criteria^4^,^7^,^8^,^9^,^10^,^11^,^12^,^13^,^14^,^15^,^16^.

The principle of potential expansion of listing criteria is to keep the risk of post-transplant tumor recurrence within the limits provided by the use of Milan criteria^6^. Accordingly, a combination of both morphological and biological tumor features seems crucial to achieve that purpose^8^. Pre-transplant serum tumor markers, namely alpha-fetoprotein (AFP) and protein induced by vitamin K absence or antagonist II, remain the most widely adopted surrogates for tumor biological aggressiveness used for that purpose^7^,^8^,^10^,^13^,^16^,^17^,^18^,^19^. However, microvascular invasion is consistently being reported as one of the most important tumor features, largely determining the long-term patient prognosis after both resection and transplantation^20^,^21^,^22^,^23^,^24^,^25^. In a recent systematic review, the relative risk for worse 3-year disease-free survival associated with microvascular invasion was 3.4 for liver transplantation and 1.8 for liver resection^26^.

Assessment of microvascular invasion on the basis of histopathological examination of the explanted liver precluded its incorporation into the selection process to date. For this reason, a large number of studies focused on the methods of pre-transplant prediction of this negative tumor feature. Due to the problem of intratumor heterogeneity, analysis of biopsy samples is inadequate for evaluation of microvascular invasion^27^. Several radiological studies have brought promising results, yet still either insufficient to provide a method of setting a definite pre-transplant diagnosis or requiring further validation^27^,^28^,^29^,^30^. Importantly, microvascular invasion is continuously reported to be associated with macroscopic tumor features, such as number and size, and serum oncologic markers^31^,^32^,^33^,^34^. Using various combinations of these factors facilitates prediction of the presence of microvascular invasion with moderate accuracy. However, significant predictors of microvascular invasion are also risk factors for post-transplant tumor recurrence^8^,^11^,^13^,^16^,^20^. Therefore, false negative results of prediction of microvascular invasion basing on other well-known factors for tumor recurrence are limited to patients at generally low risk of recurrence. Notably, microvascular invasion was found irrelevant in prediction of long-term outcomes following resection of small, therefore low risk, HCC nodules^35^.

In contrast to patients not considered for transplantation in whom information on microvascular invasion may aid the decision on choosing the appropriate treatment modality, its application in the transplant setting appears limited to potentially identify patients at high risk of recurrence to whom liver transplantation should not be offered. The aim of the present study was to evaluate the rationale for prediction of microvascular invasion prior to liver transplantation.

## Materials and Methods

A total of 1459 liver transplantations were performed in the Department of General, Transplant and Liver Surgery at the Medical University of Warsaw (Poland) in the period between December 1989 and July 2014. Between January 2003 and July 2014, there were 203 patients with HCC treated with liver transplantation. Following exclusion of 3 patients with missing data on the presence of microvascular invasion, 200 patients were included in the final study cohort. This retrospective cohort study was approved by the local ethics committee of the Medical University of Warsaw. Due to the retrospective character of the study, informed consent was not required by the institutional review board. The methods were carried out in accordance with the relevant guidelines and regulations.

Tumor recurrence over the 5-year post-transplantation period was set as the primary end-point. Recurrence-free survival, the primary outcome measure, was calculated as the time from liver transplantation to the occurrence of primary end-point. Observations were censored at the date of last available follow-up or death due to causes other than tumor recurrence. Microvascular invasion was set as the secondary end-point and defined as the presence of tumor within vessels found on microscopic evaluation and referred to in histopathologic reports. Details on surgical technique, post-transplant follow-up and immunosuppression protocol were described previously^36^,^37^.

First, predictors of the presence of microvascular invasion were established. Using the independent predictors, a score for prediction of microvascular invasion was created and an optimal cut-off value was searched for. Recurrence-free survival at 5 years post-transplantation was compared between patients with and without microvascular invasion. Moreover, the outcomes were compared between patients without microvascular invasion, patients with microvascular invasion not predicted by the established score (false negatives), and those with microvascular invasion predicted by the established score (true positives). Additionally, two previously published models were evaluated in a corresponding fashion^32^,^34^. Finally, the impact of the presence of microvascular invasion on 5-year recurrence-free survival was analyzed following adjustment for the confounding effects of the independent predictors of microvascular invasion.

Continuous variables were presented as medians with interquartile ranges. Categorical variables were presented as numbers with percentages. Logistic regression models were used to evaluate independent predictors of microvascular invasion. Forward stepwise method was used to create multivariable model with p < 0.150 used for inclusion and p < 0.050 for exclusion of variables from the model. Receiver operating characteristics (ROC) curves were used to establish the optimal cut-offs for prediction of microvascular invasion. Areas under the ROC (AUROCs) were presented with standard errors (SEs). Recurrence-free survival was calculated using the Kaplan-Meier estimator. Survival curves were compared with the log-rank test. Cox proportional hazard regression models were used to evaluate factors associated with recurrence-free survival. Odds ratios (ORs) and hazard ratios (HRs) were presented with 95% confidence intervals (95% CI). The level of significance was set at α = 0.05. STATISTICA 12 statistical software (StatSoft Inc., Tulsa, OK, USA) was used to conduct statistical analyses.

## Results

Baseline characteristics of patients included in the final study cohort were presented in Table 1. Microvascular invasion was found in 57 out of 200 patients (28.5%). Median follow-up period was 30 months. A total of 30 patients developed tumor recurrence, with the recurrence-free survival rates of 91.6% at 1 year, 83.6% at 3 years, and 79.1% at 5 years.

The following significant predictors of microvascular invasion were identified in univariable analyses: number of tumors (p < 0.001), size of the largest tumor (p = 0.004), total tumor volume (p = 0.005), pre-transplant alpha-fetoprotein concentration (p = 0.013), and poor tumor differentiation (p = 0.050, Table 2). The associations between the presence of hepatitis B virus infection (p = 0.054) and model for end-stage liver disease score (p = 0.088) and microvascular invasion were slightly above the level of significance. However, multivariable analysis revealed that only number of tumors (p = 0.001), size of the largest tumor (p = 0.009), and pre-transplant alpha-fetoprotein concentration (p = 0.049) were independent predictors of microvascular invasion.

**Figure 1:**
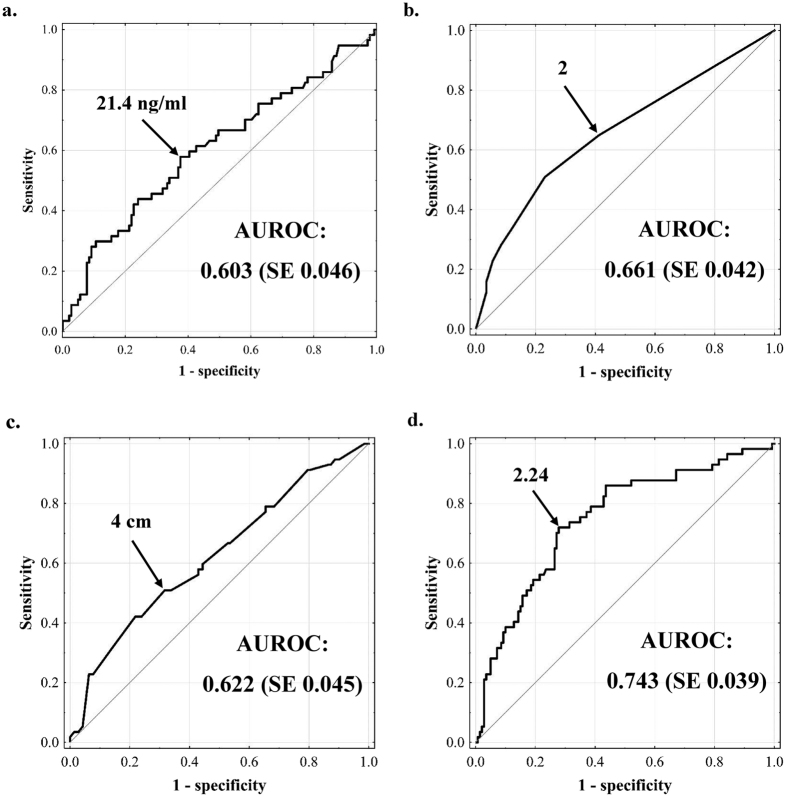
Assessment of the optimal variables cut-offs in prediction of microvascular invasion. Receiver operating characteristics curve for pre-transplant alpha-fetoprotein concentration (**a**), number of tumors (**b**), size of the largest tumor (**c**), and microvascular invasion index (**d**) in prediction of microvascular invasion. AUROC – area under the receiver operating characteristics curve; SE – standard error.

Prediction of microvascular invasion based on pre-transplant alpha-fetoprotein concentration, number of tumors, and size of the largest tumor was associated with AUROCs of 0.603 (SE = 0.046), 0.661 (SE = 0.042), and 0.622 (SE = 0.045), respectively (Fig. 1a,b, and c). According to the ROC curves, the optimal cut-offs were: ≥21.4 ng/ml for pre-transplant alpha-fetoprotein concentration, ≥2 for number of tumors, and ≥4 cm for size of the largest tumor. The established cut-offs were associated with accuracy, sensitivity, specificity, positive predictive value, and negative predictive value of 61.1%, 57.9%, 62.4%, 38.4%, and 78.6%, respectively, for pre-transplant alpha-fetoprotein, 60.5%, 64.9%, 58.7%, 38.5%, and 80.8%, respectively, for number of tumors, and 63.3%, 50.9%, 68.3%, 39.2%, and 77.6%, respectively, for size of the largest tumor.

Microvascular invasion index (MVI index) was created basing on the results of multivariable analysis of the associations between number of tumors, size of the largest tumor and pre-transplant alpha-fetoprotein concentration and microvascular invasion, and was defined as:

MVI index  =  0.293 x (number of tumors) + 0.283 x (size of the largest tumor in cm) + 0.164 x log_e_(pre-transplant alpha-fetoprotein concentration in ng/ml).

The AUROC for prediction of microvascular invasion based on MVI index (Fig. 1d) was 0.743 (SE 0.039), significantly higher than each of those observed for the three independent predictors: pre-transplant alpha-fetoprotein (p = 0.002), number of tumors (p = 0.022), and size of the largest tumor (p = 0.001). As compared to the MVI index, the use of either of the analyzed, previously published models was associated with non-significantly lower AUROC (Table 3). Accuracy, sensitivity, specificity, positive predictive value and negative predictive value for the established MVI index cut-off value of 2.24 were 72.1%, 71.9%, 72.1%, 51.3%, and 86.3%, respectively.

**Figure 2:**
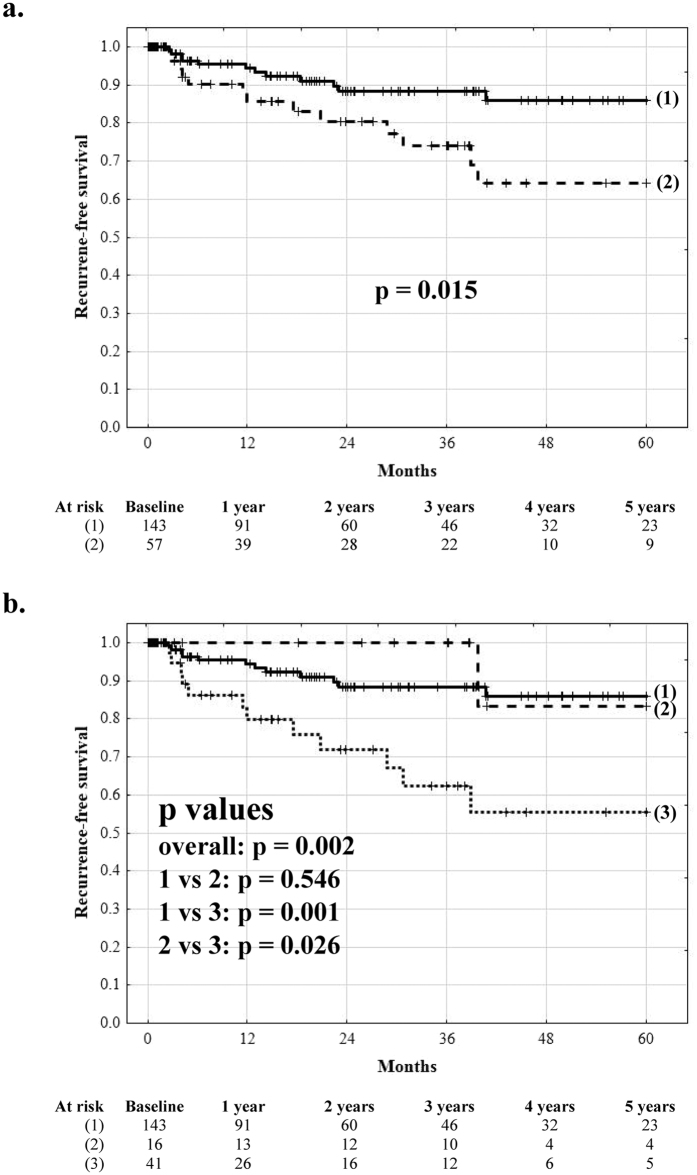
The impact of low- and high-risk microvascular invasion on outcomes after liver transplantation. Recurrence-free survival curves after liver transplantation in (**a**) patients with (dashed line) and without (solid line) microvascular invasion, and (**b**) in patients without microvascular invasion (solid line), patients with microvascular invasion unpredicted by the model (dashed line), and patients with microvascular invasion predicted by the model (dotted line). Numbers of patients at risk are presented below the graphs.

**Figure 3:**
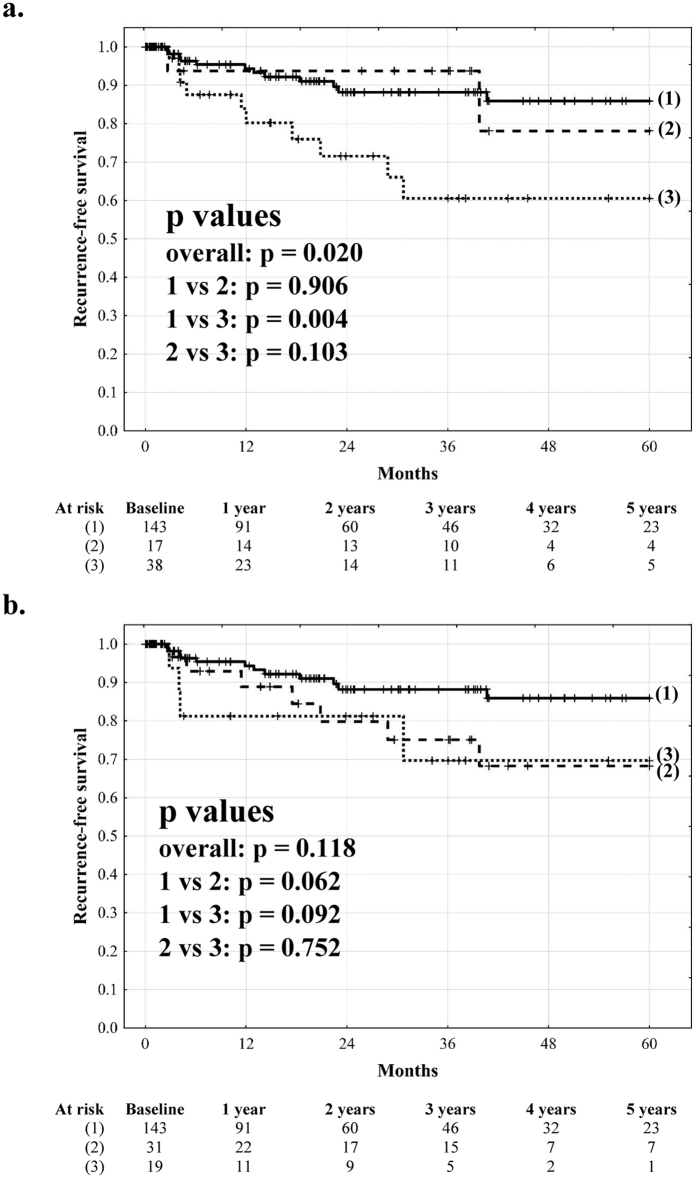
The impact of microvascular invasion predicted and unpredicted by the scores proposed by Cucchetti *et al*.^34^ and Zhao *et al*.^32^ on outcomes after liver transplantation. Recurrence-free survival curves after liver transplantation in (**a**) patients without microvascular invasion (solid line), patients with microvascular invasion unpredicted by the model proposed by Cucchetti *et al*. (dashed line), and patients with microvascular invasion predicted by the model (dotted line) and (**b**) in patients without microvascular invasion (solid line), patients with microvascular invasion unpredicted by the model proposed by Zhao *et al*. (dashed line), and patients with microvascular invasion predicted by the model (dotted line). Numbers of patients at risk are presented below the graphs.

Recurrence-free survival at 1, 3, and 5 years post-transplantation was 85.6%, 74.0%, and 64.1%, respectively, in patients with microvascular invasion, significantly inferior to that of 94.3%, 88.3%, and 85.9%, respectively, observed in patients without microvascular invasion (p = 0.015; Fig. 2a). Patients with microvascular invasion not predicted by the MVI index (MVI index  < 2.24) exhibited 5-year recurrence free survival of 83.3%, superior to 55.3% (p = 0.026) observed in those with microvascular invasion predicted by the MVI index (MVI index ≥2.24). As compared to patients without microvascular invasion, patients with microvascular invasion predicted by the MVI index (true positive prediction) had significantly lower (p = 0.001) while patients with microvascular invasion not predicted by the MVI index (false negative prediction) similar (p = 0.546) 5-year recurrence free survival (Fig. 2b). Similarly, patients with false negative and true positive prediction of microvascular invasion based on the Cucchetti *et al* score had similar (78.1%) and significantly lower (60.6%) 5-year recurrence free survival, respectively, than patients without microvascular invasion (p = 0.906 and p = 0.004, respectively, Fig. 3a). Conversely, patients with either microvascular invasion predicted or unpredicted by the Zhao *et al* score exhibited non-significantly (p = 0.092 and p = 0.062, respectively) compromised recurrence-free survival at 5 years (69.6% and 68.3%, respectively, Fig. 3b).

Microvascular invasion (p = 0.021), number of tumors (p < 0.001), size of the largest tumor (p < 0.001), total tumor volume (p < 0.001), pre-transplant alpha-fetoprotein concentration (p < 0.001), and poor tumor differentiation (p = 0.005) were significantly associated with 5-year recurrence-free survival in univariable analyses (Table 4). Number of tumors (p = 0.047), size of the largest tumor (p < 0.001), pre-transplant alpha-fetoprotein (p < 0.001), and poor tumor differentiation (p = 0.039) were independent risk factors for HCC recurrence in multivariable analysis. No significant impact of microvascular invasion on 5-year recurrence-free survival was found following adjustment for the effects of number of tumors, size of the largest tumor, and pre-transplant alpha-fetoprotein in a 4-variable model (HR 1.56 95% CI 0.66–3.65; p = 0.307). Moreover, the effects of microvascular were non-significant following separate adjustment for number of tumors (microvascular invasion: HR 1.72 95% CI 0.74–3.97, p = 0.206; number of tumors: HR per 1 tumor increase 1.24 95% CI 1.09–1.40, p < 0.001), size of the largest tumor (microvascular invasion: HR 2.17 95% CI 0.95–4.99, p = 0.067; size of the largest tumor: HR per 1 cm increase 1.34 95% CI 1.15–1.57, p < 0.001), and pre-transplant alpha-fetoprotein concentration (microvascular invasion: HR 2.08 95% CI 0.91–4.76, p = 0.082; alpha-fetoprotein: HR per 1 log_e_ increase 1.39 95% CI 1.20–1.61, p < 0.001) in bivariable analyses, while the independent risk factors retained their significant effects.

## Discussion

Microvascular invasion is frequently reported as a major risk factor for HCC recurrence following liver transplantation^20^,^38^,^39^. As such, in order to facilitate its inclusion into the selection process, a wide variety of studies are focused on optimizing methods of preoperative prediction of microvascular invasion using imaging modalities and other pre-transplant factors^27^,^28^,^29^,^30^,^40^,^41^,^42^. The results of the present study confirm that a combination of morphological tumor characteristics with serum alpha-fetoprotein concentration may be used for this purpose with moderate accuracy. However, what is more important, the findings indicate that pre-transplant assessment of microvascular invasion does not appear to provide additional information on the risk of post-transplant tumor recurrence when both morphological and biological features are included in the selection criteria.

Recurrence-free survival of patients with microvascular invasion was indeed inferior to those without microvascular invasion, which is in line with previous reports^20^,^38^,^39^. Notably, the negative effect of microvascular invasion was only slightly above the level of significance following separate adjustment for the effects of size of the largest tumor and pre-transplant alpha-fetoprotein concentration. However, when all of the 3 predictors of microvascular invasion along with microvascular invasion itself were included in a single model, no significant effects of the latter were observed. Therefore, as combination of morphological tumor parameters and serum HCC markers in the selection process is gaining increasing popularity among transplant centers^7^,^8^,^10^,^13^,^16^,^17^,^18^,^19^, the relevance of obtaining data on the presence of microvascular invasion appears to become far less important for the clinical practice. This observation corresponds to previous findings of other authors regarding no significant impact of microvascular invasion in multivariable analyses including these important features^43^,^44^,^45^.

Notably, a model based on number of tumors, size of the largest tumor and pre-transplant alpha-fetoprotein concentration did not only allow to predict a major proportion of patients with microvascular invasion, but also stratified patients with microvascular invasion into those with low and high risk of tumor recurrence. Moreover, patients with low-risk microvascular invasion had their oncological outcomes almost identical to those without any microvascular invasion, which further supports the hypothesis that the use of combined morphological and biological selection criteria alleviates the need for preoperative diagnosis of microvascular invasion. This is partly in line with a recently reported lack of negative impact of microvascular invasion on long-term survival of low risk HCC patients undergoing curative resection^35^. The concept of division of patients with microvascular invasion with and without clinical relevance was further explored in a recent study by Iguchi *et al*.^39^. Basing on histopathological features, the authors of that study divided patients into those with high and low risk microvascular invasion. Importantly, the recurrence-free survival curves of patients with low and high risk microvascular invasion in the Iguchi *et al*. study highly resembled those observed in the present study for patients with microvascular invasion unpredicted and predicted by the MVI index, respectively.

Given that a model based solely on preoperative factors was able to effectively stratify patients with microvascular invasion into those with low and high risk of tumor recurrence, the results of this study partly oppose the role of liver resection in selection of patients with HCC for liver transplantation. As patients with microvascular invasion are at higher risk of recurrence in general, diagnosis of microvascular invasion in resected specimens was proposed as an exclusion criterion for liver transplantation^46^. Notably, a potential role of alpha-fetoprotein concentration in identification of patients with microvascular invasion and unresectable tumors was also acknowledged by the same authors. The results of the present study clearly point towards a more profound role of this marker in selection of patients for liver transplantation, as useful in prediction of high-risk microvascular invasion when combined with morphological parameters. Conversely, the concept of *ab initio* transplantation after liver resection in patients with a diagnosis of microvascular invasion in surgical specimen was suggested as an alternate strategy in order to maximize the benefit of liver transplantation over resection in the era of organ shortage^47^. Its feasibility was recently reported, yet the use of an additional criterion of a minimum 6-month recurrence-free interval was suggested to avoid early recurrences^48^. Accordingly, the results of this study indicate that such additional criterion may be replaced with preoperative stratification of patients into those with low and high risk microvascular invasion. On the other hand, the follow-up period after liver resection provides a unique opportunity to assess the actual tumor biological aggressiveness^49^. Whether preoperative assessment is comparably accurate remains to be elucidated, yet available data on generally negative outcomes after liver resection combined with the option of salvage liver transplantation on intention-to-treat basis also need to be considered^50^.

The general characteristics of the created model in prediction of microvascular invasion are lower than other previously published^32^,^33^,^34^. However, application of either the score proposed by Cucchetti *et al*. or that proposed by Zhao *et al*. to patients included in the present study was associated with non-significantly lower AUROCs as compared to the MVI index, remarkably lower than reported in the original studies^32^,^34^. Moreover, the two previously published prediction models differed with respect to the clinical significance of false negative results. In line with the MVI index, patients with microvascular invasion predicted by the score proposed by Cucchetti *et al*. exhibited significantly compromised recurrence-free survival, whereas patients with microvascular invasion “missed” by the score exhibited outcomes similar to patients without microvascular invasion. In contrast, the score proposed by Zhao *et al*. lacked the ability to stratify patients with microvascular invasion into those at low and high risk of post-transplant HCC recurrence. This may be due to inclusion of the γ-glutamyl-transpeptidase activity in the latter, as both the MVI index and the score proposed by Cucchetti *et al*. are based only on variables known to be associated with tumor burden or biological behavior.

Notably, recently proposed detailed analyses of imaging studies appear more accurate with positive predictive value exceeding 90%^30^. The artificial neural network method introduced by Cucchetti *et al*. along a separate logistic regression model was reported to predict microvascular invasion with similar precision^34^. A score proposed by Shirabe *et al*. based on tumor size, des-gamma-carboxy prothrombin concentration and maximum standardized uptake value on 2-[18 F]-fluoro-2-deoxy-D-glucose positron emission tomography also provided superior sensitivity and specificity rates of 100% and 91%, respectively^33^. Unfortunately, the data available in the present study were insufficient to validate that previous findings. However, the most important advantage of combining morphological tumor characteristics with serum alpha-fetoprotein concentration is to provide data on high-risk, rather than to identify all patients with microvascular invasion, and the presented model was created solely to evaluate such capability. Therefore, the use of highly sensitive and specific predictive models in the selection process would lead to exclusion of patients with low-risk microvascular invasion from liver transplantation.

According to a report from an international consensus conference, microvascular invasion was not recommended for inclusion in the process of selection of patients for liver transplantation^6^. While the recommendation was based on the lack of reliable methods of pre-transplant detection, the results of the present study point towards the lack of prognostic significance of microvascular invasion when both morphological and biological criteria are considered and thus, support the guidelines for a different reason.

The study is subject to the limitation of its retrospective nature. Moreover, given the number of patients, there is a risk of type II error in the assessment of the effects of microvascular invasion. On the other hand, the observed outcomes of patients with low-risk microvascular invasion seem to preclude any clinically relevant inferiority as compared to patients without microvascular invasion.

In conclusion, the results of this study indicate that preoperative variables may be used to predict microvascular invasion with moderate accuracy. More importantly, their combination facilitates identification of patients with microvascular invasion of high-risk of tumor recurrence. Provided that morphological and biological criteria are combined in the selection of patients for liver transplantation, diagnosis of microvascular invasion does not seem to aid the decision-making processes and thus, appears unnecessary prior to transplantation.

## Additional Information

**How to cite this article**: Grąt, M. *et al*. Limitations of predicting microvascular invasion in patients with hepatocellular cancer prior to liver transplantation. *Sci. Rep.* **7**, 39881; doi: 10.1038/srep39881 (2017).

**Publisher's note:** Springer Nature remains neutral with regard to jurisdictional claims in published maps and institutional affiliations.

## Figures and Tables

**Table 1:** Baseline characteristics of 200 patients after liver transplantation for hepatocellular cancer included in the final study cohort.

Characteristics	n (%) or median (IQR)
Recipient gender	
male	143 (71.5%)
female	57 (28.5%)
Recipient age (years)	57 (52–61)
MELD	11 (8–13)
HCV infection	137 (68.5%)
HBV infection	81 (40.5%)
Within Milan criteria	120 (60.0%)
Within UCSF criteria	144 (72.0%)
Within Up-to-7 criteria	154 (77.0%)
Number of tumors	1 (1–3)
Size of the largest tumor (cm)	3.0 (2.0–4.5)
Total tumor volume (cm^3^)	22.5 (5.3–54.2)
Pre-transplant AFP (ng/ml)	16 (6–114)
Poor tumor differentiation	24 (12.0%)
Microvascular invasion	57 (28.5%)
Neoadjuvant treatment	86 (43.0%)
Total ischemic time (hours)	9.0 (8.0–10.3)
Piggyback transplantations	175 (87.5%)
Intraoperative PRBC transfusions (units)	3.0 (1.5–6.0)
Intraoperative FFP transfusions (units)	7.0 (5.0–10.0)
Donor age (years)	49.5 (38.0–57.5)

IQR – interquartile range; UCSF – University of California, San Francisco; AFP – alpha-fetoprotein; MELD – model for end-stage liver disease; HCV – hepatitis C virus; HBV – hepatitis B virus; PRBC – packed red blood cells; FFP – fresh frozen plasma.

**Table 2:** Results of the analyses of predictors of the presence of microvascular invasion in patients with hepatocellular cancer undergoing liver transplantation.

Factors	Univariable		Multivariable	
	OR (95% CI)	p	OR (95% CI)	p
Recipient male gender	1.02 (0.72–1.43)	0.932		
Recipient age	1.03 (0.99–1.07)	0.192		
MELD	0.94 (0.88–1.01)	0.088		
HCV infection	0.84 (0.61–1.17)	0.306		
HBV infection	0.72 (0.52–1.01)	0.054		
Alcoholic liver disease	1.31 (0.89–1.92)	0.177		
Number of tumors	1.28 (1.12–1.46)	<.001	1.34 (1.13–1.59)	0.001
Size of the largest tumor	1.31 (1.09–1.56)	0.004	1.33 (1.07–1.64)	0.009
Total tumor volume	1.08 (1.02–1.15)	0.005		
Pre-transplant AFP	1.20 (1.04–1.38)	0.013	1.18 (1.01–1.39)	0.049
Poor tumor differentiation	1.55 (1.01–2.39)	0.050		
Neoadjuvant treatment	1.16 (0.63–2.15)	0.638		

Odds ratios were given per: 1 year increase for recipient age; 1 point increase for model for end-stage liver disease; 1 tumor more for number of tumors; 1 cm increase for the size of the largest tumor; 10 cm^3^ increase for total tumor volume; 1 log_e_ increase for alpha-fetoprotein. OR – odds ratio; 95% CI – 95% confidence interval; MELD – model for end-stage liver disease; HCV – hepatitis C virus; HBV – hepatitis B virus; AFP – alpha-fetoprotein.

**Table 3:** Characteristics of the MVI index and two previously published scores in prediction of microvascular invasion in patients with hepatocellular cancer undergoing liver transplantation.

Score	Prediction of microvascular invasion							
	AUROC (SE)	AUROC (reported previously)	Cut-off	Accuracy	Sensitivity	Specificity	PPV	NPV
MVI index	0.743 (0.039)	—	≥2.24	72.1%	71.9%	72.1%	51.3%	86.3%
Cucchetti *et al*.^34^	0.690^a^ (0.041)	0.850^34^	≥3.407	66.3%	69.1%	65.2%	44.7%	83.8%
Zhao *et al*.^32^	0.674^b^ (0.039)	0.832^32^	≥3	77.5%	38.0%	93.5%	70.4%	78.8%

MVI index = 0.293 x (number of tumors) + 0.283 x (size of the largest tumor in cm) + 0.164 x loge(pre-transplant alpha-fetoprotein concentration in ng/ml)

Cucchetti *et al*. score^34^ = −5.087 + 2.417 x log_10_(pre-transplant alpha-fetoprotein concentration in ng/ml) + 0.778 x (size of the largest tumor in cm) + 1.550 x log_10_(total tumor volume in cm^3^)

Zhao *et al* score^32^ = 1 point if pre-transplant alpha-fetoprotein concentration > 400 μg/L + 2 points if pre-transplant γ-glutamyl-transpeptidase activity > 130 U/L + 1 point if total tumor size > 8 cm + 2 if >3 tumors

a – p = 0.062 as compared to MVI index; b – p = 0.104 as compared to MVI index

AUROC – area under the receiver operating characteristics curve; SE – standard error; PPV – positive predictive value; NPV – negative predictive value

**Table 4:** Results of the analyses of factors associated with 5-year recurrence-free survival after liver transplantation for hepatocellular cancer.

Factors	Univariable		Multivariable	
	HR (95% CI)	p	HR (95% CI)	p
Recipient male gender	1.21 (0.45–3.23)	0.701		
Recipient age	0.98 (0.94–1.02)	0.380		
MELD	0.95 (0.86–1.06)	0.361		
HCV infection	0.80 (0.36–1.78)	0.583		
HBV infection	1.80 (0.82–3.95)	0.142		
Alcoholic liver disease	1.08 (0.40–2.88)	0.879		
Number of tumors	1.27 (1.13–1.43)	<.001	1.18 (1.01–1.38)	0.047
Size of the largest tumor	1.39 (1.19–1.62)	<.001	1.33 (1.13–1.56)	<.001
Total tumor volume	1.03 (1.01–1.04)	<.001		
Pre-transplant AFP	1.43 (1.23–1.66)	<0.001	1.45 (1.20–1.75)	<.001
Poor tumor differentiation	3.53 (1.47–8.48)	0.005	2.95 (1.05–8.25)	0.039
Microvascular invasion	2.52 (1.15–5.52)	0.021		
Neoadjuvant treatment	1.82 (0.83–4.01)	0.136		
Donor age (years)	0.99 (0.96–1.02)	0.573		

Hazard ratios were given per: 1 year increase for recipient and donor age; 1 point increase for model for end-stage liver disease; 1 tumor more for number of tumors; 1 cm increase for the size of the largest tumor; 10 cm^3^ increase for total tumor volume; 1 log_e_ increase for alpha-fetoprotein. HR – hazard ratio; 95% CI – 95% confidence interval; MELD – model for end-stage liver disease; HCV – hepatitis C virus; HBV – hepatitis B virus; AFP – alpha-fetoprotein.

## References

[b1] Bruix J., Gores G. J. & Mazzaferro V. Hepatocellular carcinoma: clinical frontiers and perspectives. Gut 63, 844–855 (2014). 24531850 10.1136/gutjnl-2013-306627 PMC4337888

[b2] Dutkowski P., Linecker M., DeOliveira M. L., Müllhaupt B. & Clavien P. A. Challenges to liver transplantation and strategies to improve outcomes. Gastroenterology 148, 307–323 (2015). 25224524 10.1053/j.gastro.2014.08.045

[b3] Geissler E. K. . Sirolimus Use in Liver Transplant Recipients With Hepatocellular Carcinoma: A Randomized, Multicenter, Open-Label Phase 3 Trial. Transplantation 100, 116–125 (2016). 26555945 10.1097/TP.0000000000000965 PMC4683033

[b4] Sapisochin G. . The extended toronto criteria for liver transplantation in patients with hepatocellular carcinoma: A prospective validation study. Hepatology 10.1002/hep.28643 (2016). 27178646

[b5] Piñero F. . Liver Transplantation for Hepatocellular carcinoma: Evaluation of the AFP model in a multicenter cohort from Latin America. Liver Int. 10.1111/liv.13159 (2016). 27169841

[b6] Clavien P. A. . Recommendations for liver transplantation for hepatocellular carcinoma: an international consensus conference report. Lancet Oncol. 13, e11–22 (2012). 22047762 10.1016/S1470-2045(11)70175-9 PMC3417764

[b7] Toso C. . Total tumor volume and alpha-fetoprotein for selection of transplant candidates with hepatocellular carcinoma: A prospective validation. Hepatology 62, 158–165 (2015). 25777590 10.1002/hep.27787

[b8] Grąt M. . Combination of morphologic criteria and α-fetoprotein in selection of patients with hepatocellular carcinoma for liver transplantation minimizes the problem of posttransplant tumor recurrence. World J. Surg. 38, 2698–2707 (2014). 24858191 10.1007/s00268-014-2647-3 PMC4161934

[b9] Balci D. . Living donor liver transplantation for hepatocellular carcinoma: a single center analysis of outcomes and impact of different selection criteria. Transpl. Int. 24, 1075–1083 (2011). 21854454 10.1111/j.1432-2277.2011.01311.x

[b10] Duvoux C. . Liver transplantation for hepatocellular carcinoma: a model including α-fetoprotein improves the performance of Milan criteria. Gastroenterology 143, 986–994 (2012). 22750200 10.1053/j.gastro.2012.05.052

[b11] Mazzaferro V. . Predicting survival after liver transplantation in patients with hepatocellular carcinoma beyond the Milan criteria: a retrospective, exploratory analysis. Lancet Oncol. 10, 35–43 (2009). 19058754 10.1016/S1470-2045(08)70284-5

[b12] Yao F. Y. . Liver transplantation for hepatocellular carcinoma: validation of the UCSF-expanded criteria based on preoperative imaging. Am. J. Transplant. 7, 2587–2596 (2007). 17868066 10.1111/j.1600-6143.2007.01965.x

[b13] Takada Y. . Living donor liver transplantation for patients with HCC exceeding the Milan criteria: a proposal of expanded criteria. Dig. Dis. 25, 299–302 (2007). 17960063 10.1159/000106908

[b14] Lee S. G. . Expanded indication criteria of living donor liver transplantation for hepatocellular carcinoma at one large-volume center. Liver Transpl. 14, 935–945 (2008). 18581465 10.1002/lt.21445

[b15] Sugawara Y., Tamura S. & Makuuchi M. Living donor liver transplantation for hepatocellular carcinoma: Tokyo University series. Dig. Dis. 25, 310–312 (2007). 17960065 10.1159/000106910

[b16] Zheng S. S. . Liver transplantation for hepatocellular carcinoma: Hangzhou experiences. Transplantation 85, 1726–1732 (2008). 18580463 10.1097/TP.0b013e31816b67e4

[b17] Chaiteerakij R. . Combinations of biomarkers and Milan criteria for predicting hepatocellular carcinoma recurrence after liver transplantation. Liver Transpl. 21, 599–606 (2015). 25789635 10.1002/lt.24117 PMC4490162

[b18] Shindoh J. . Evaluation methods for pretransplant oncologic markers and their prognostic impacts in patient undergoing living donor liver transplantation for hepatocellular carcinoma. Transpl. Int. 27, 391–398 (2014). 24472068 10.1111/tri.12274

[b19] Fujiki M. . Significance of des-gamma-carboxy prothrombin in selection criteria for living donor liver transplantation for hepatocellular carcinoma. Am. J. Transplant. 9, 2362–2371 (2009). 19656125 10.1111/j.1600-6143.2009.02783.x

[b20] Agopian V. G. . A novel prognostic nomogram accurately predicts hepatocellular carcinoma recurrence after liver transplantation: analysis of 865 consecutive liver transplant recipients. J. Am. Coll. Surg. 220, 416–427 (2015). 25690672 10.1016/j.jamcollsurg.2014.12.025

[b21] Kluger M. D. . Liver resection for hepatocellular carcinoma in 313 Western patients: tumor biology and underlying liver rather than tumor size drive prognosis. J. Hepatol. 62, 1131–1140 (2015). 25529622 10.1016/j.jhep.2014.12.018

[b22] Shim J. H. . Prognostic nomograms for prediction of recurrence and survival after curative liver resection for hepatocellular carcinoma. Ann. Surg. 261, 939–946 (2015). 24950276 10.1097/SLA.0000000000000747

[b23] He J. . The Clinicopathologic and Prognostic Significance of Gross Classification on Solitary Hepatocellular Carcinoma After Hepatectomy. Medicine (Baltimore) 94, e1331 (2015). 26266378 10.1097/MD.0000000000001331 PMC4616685

[b24] Hou Y. F. . Second Hepatectomy Improves Survival in Patients With Microvascular Invasive Hepatocellular Carcinoma Meeting the Milan Criteria. Medicine (Baltimore) 94, e2070 (2015). 26632890 10.1097/MD.0000000000002070 PMC4674193

[b25] Yan X. . Infiltrative Hepatocellular Carcinoma: Assessment of Factors Associated With Outcomes in Patients Undergoing Hepatectomy. Medicine (Baltimore) 95, e3589 (2016). 27175659 10.1097/MD.0000000000003589 PMC4902501

[b26] Rodríguez-Perálvarez M. . A systematic review of microvascular invasion in hepatocellular carcinoma: diagnostic and prognostic variability. Ann. Surg. Oncol. 20, 325–339 (2013). 23149850 10.1245/s10434-012-2513-1

[b27] Banerjee S. . A computed tomography radiogenomic biomarker predicts microvascular invasion and clinical outcomes in hepatocellular carcinoma. Hepatology 62, 792–800 (2015). 25930992 10.1002/hep.27877 PMC4654334

[b28] Kobayashi T. . Preoperative Fluorine 18 Fluorodeoxyglucose Positron Emission Tomography/Computed Tomography for Prediction of Microvascular Invasion in Small Hepatocellular Carcinoma. J. Comput. Assist. Tomogr. 40, 524–530 (2016). 26966955 10.1097/RCT.0000000000000405

[b29] Wu T. H. . A non-smooth tumor margin on preoperative imaging predicts microvascular invasion of hepatocellular carcinoma. Surg. Today 10.1007/s00595-016-1320-x (2016). 26983710

[b30] Renzulli M. . Can Current Preoperative Imaging Be Used to Detect Microvascular Invasion of Hepatocellular Carcinoma? Radiology 279, 432–442 (2016). 26653683 10.1148/radiol.2015150998

[b31] Poté N. . Performance of PIVKA-II for early hepatocellular carcinoma diagnosis and prediction of microvascular invasion. J. Hepatol. 62, 848–854 (2015). 25450201 10.1016/j.jhep.2014.11.005

[b32] Zhao W. C. . Preoperative predictors of microvascular invasion in multinodular hepatocellular carcinoma. Eur. J. Surg. Oncol. 39, 858–864 (2013). 23669199 10.1016/j.ejso.2013.04.003

[b33] Shirabe K. . New scoring system for prediction of microvascular invasion in patients with hepatocellular carcinoma. Liver Int. 34, 937–941 (2014). 24393295 10.1111/liv.12459

[b34] Cucchetti A. . Preoperative prediction of hepatocellular carcinoma tumour grade and micro-vascular invasion by means of artificial neural network: a pilot study. J. Hepatol. 52, 880–888 (2010). 20409605 10.1016/j.jhep.2009.12.037

[b35] Shindoh J. . Microvascular invasion does not predict long-term survival in hepatocellular carcinoma up to 2 cm: reappraisal of the staging system for solitary tumors. Ann. Surg. Oncol. 20, 1223–1229 (2013). 23179993 10.1245/s10434-012-2739-y PMC3856190

[b36] Grąt M. . The impact of surgical technique on the results of liver transplantation in patients with hepatocellular carcinoma. Ann. Transplant. 18, 448–459 (2013). 24008493 10.12659/AOT.884005

[b37] Krawczyk M. . 1000 liver transplantations at the Department of General, Transplant and Liver Surgery, Medical University of Warsaw--analysis of indications and results. Pol. Przegl. Chir. 84, 304–312 (2012). 22842743 10.2478/v10035-012-0051-y

[b38] Marques H. P. . Long-term Results of Domino Liver Transplantation for Hepatocellular Carcinoma Using the “Double Piggy-back” Technique: A 13-Year Experience. Ann. Surg. 262, 749–756 (2015). 26583662 10.1097/SLA.0000000000001446

[b39] Iguchi T. . New Pathologic Stratification of Microvascular Invasion in Hepatocellular Carcinoma: Predicting Prognosis After Living-donor Liver Transplantation. Transplantation 99, 1236–1242 (2015). 25427164 10.1097/TP.0000000000000489

[b40] Ahn S. Y. . Prediction of microvascular invasion of hepatocellular carcinoma using gadoxetic acid-enhanced MR and (18)F-FDG PET/CT. Abdom. Imaging 40, 843–851 (2015). 25253426 10.1007/s00261-014-0256-0

[b41] Wu D. . Liver computed tomographic perfusion in the assessment of microvascular invasion in patients with small hepatocellular carcinoma. Invest. Radiol. 50, 188–194 (2015). 25333306 10.1097/RLI.0000000000000098

[b42] Min J. H. . Prediction of microvascular invasion of hepatocellular carcinomas with gadoxetic acid-enhanced MR imaging: Impact of intra-tumoral fat detected on chemical-shift images. Eur. J. Radiol. 84, 1036–1043 (2015). 25818729 10.1016/j.ejrad.2015.03.002

[b43] Schraiber Ldos S. . Alpha-fetoprotein Level Predicts Recurrence After Transplantation in Hepatocellular Carcinoma. Medicine (Baltimore) 95, e2478 (2016). 26817881 10.1097/MD.0000000000002478 PMC4998255

[b44] Miltiadous O. . Progenitor cell markers predict outcome of patients with hepatocellular carcinoma beyond Milan criteria undergoing liver transplantation. J. Hepatol. 63, 1368–1377 (2015). 26220754 10.1016/j.jhep.2015.07.025 PMC12182650

[b45] Zhang Q. . α-Fetoprotein is a potential survival predictor in hepatocellular carcinoma patients with hepatitis B selected for liver transplantation. Eur. J. Gastroenterol. Hepatol. 26, 544–552 (2014). 24614696 10.1097/MEG.0000000000000029

[b46] Piardi T. . Number and tumor size are not sufficient criteria to select patients for liver transplantation for hepatocellular carcinoma. Ann. Surg. Oncol. 19, 2020–2026 (2012). 22179632 10.1245/s10434-011-2170-9

[b47] Sala M. . High pathological risk of recurrence after surgical resection for hepatocellular carcinoma: an indication for salvage liver transplantation. Liver Transpl. 10, 1294–1300 (2004). 15376311 10.1002/lt.20202

[b48] Ferrer-Fàbrega J. . Prospective validation of ab initio liver transplantation in hepatocellular carcinoma upon detection of risk factors for recurrence after resection. Hepatology 63, 839–849 (2016). 26567038 10.1002/hep.28339

[b49] Lee H. S. . The clinical behavior of transplantable recurrent hepatocellular carcinoma after curative resection: implications for salvage liver transplantation. Ann. Surg. Oncol. 21, 2717–2724 (2014). 24916744 10.1245/s10434-014-3597-6

[b50] Liu F. . Salvage liver transplantation for recurrent hepatocellular carcinoma within UCSF criteria after liver resection. PLoS One 7, e48932 (2012). 23145027 10.1371/journal.pone.0048932 PMC3493590

